# Propensity score interval matching: using bootstrap confidence intervals for accommodating estimation errors of propensity scores

**DOI:** 10.1186/s12874-015-0049-3

**Published:** 2015-07-28

**Authors:** Wei Pan, Haiyan Bai

**Affiliations:** School of Nursing, Duke University, DUMC 3322, 307 Trent Drive, Durham, NC 27710 USA; Department of Educational and Human Sciences, University of Central Florida, PO Box 161250, Orlando, FL 32816 USA

**Keywords:** Observational studies, Propensity score methods, Propensity score matching, Nearest neighbour matching, Caliper matching, The bootstrap, Confidence intervals, Causal inference

## Abstract

**Background:**

Propensity score methods have become a popular tool for reducing selection bias in making causal inference from observational studies in medical research. Propensity score matching, a key component of propensity score methods, normally matches units based on the distance between point estimates of the propensity scores. The problem with this technique is that it is difficult to establish a sensible criterion to evaluate the closeness of matched units without knowing estimation errors of the propensity scores.

**Methods:**

The present study introduces *interval matching* using bootstrap confidence intervals for accommodating estimation errors of propensity scores. In interval matching, if the confidence interval of a unit in the treatment group overlaps with that of one or more units in the comparison group, they are considered as matched units.

**Results:**

The procedure of interval matching is illustrated in an empirical example using a real-life dataset from the Nursing Home Compare, a national survey conducted by the Centers for Medicare and Medicaid Services. The empirical example provided promising evidence that interval matching reduced more selection bias than did commonly used matching methods including the rival method, caliper matching. Interval matching’s approach methodologically sounds more meaningful than its competing matching methods because interval matching develop a more “scientific” criterion for matching units using confidence intervals.

**Conclusions:**

Interval matching is a promisingly better alternative tool for reducing selection bias in making causal inference from observational studies, especially useful in secondary data analysis on national databases such as the Centers for Medicare and Medicaid Services data.

**Electronic supplementary material:**

The online version of this article (doi:10.1186/s12874-015-0049-3) contains supplementary material, which is available to authorized users.

## Background

Observational studies are common in medical research because of practical or ethical barriers to random assignment of units (e.g., patients) into treatment conditions (e.g., treatment vs. comparison); consequently, observational studies likely yield results with limited validity for causal inference due to selection bias resulted from non-randomization. To reduce selection bias, Rosenbaum and Rubin [1] proposed propensity score methods for balancing the distributions of observed covariates between treatment conditions and, therefore, approximating a situation that is normally achieved through randomization.

A propensity score is defined as the probability of a unit being assigned to the treatment group [1]. Propensity score methods normally comprise four major steps [2]:

1. Estimate a propensity score for each unit using a logistic regression of treatment conditions on covariates or other propensity score estimation methods [2, 3];
2. Match each unit in the treatment group with one or more units in the comparison group based on the closest distance between their propensity scores;
3. Evaluate the matching quality in terms of how much selection bias is reduced; and
4. Conduct intended outcome analysis on the matched data or on the original data with propensity score adjustment or weighting.

Although propensity score methods have become increasingly popular in medical research over the past three decades as an effective tool for reducing selection bias in making causal inference based on observational data, propensity score matching (PSM), as a crucial step in propensity score methods, still has limitations [2]. For example, in the existent PSM techniques, matching is done primarily based on the distance between *point estimates* of propensity scores, and thus, it is difficult to establish a meaningful criterion to evaluate the closeness of the matched units without knowing the estimation errors (or standard errors) of the estimated propensity scores. Previously, Cochran and Rubin [4] proposed caliper matching, which uses a caliper band (e.g., a pre-specified distance between propensity scores) to avoid “bad” matches that are not close enough. Unfortunately, a caliper band is expressed as a proportion to the pooled standard deviation of propensity scores across all the units, and therefore, it is *unit-invariant*; that is, a caliper band takes the same value for all the units. Therefore, a caliper band does not possess a feature that can gauge the *unit-specific* standard error of the estimated propensity score for each individual unit.

The purpose of the present study was to extend caliper matching to a new matching technique, *interval matching*, by using unit-specific bootstrap confidence intervals (CIs) [5] for gauging the standard error of the estimated propensity score for each unit. In interval matching, if the confidence interval of a unit in the treatment group overlaps with that of one or more units in the comparison group, they are considered as matched units. In the present study, the procedure of interval matching is illustrated in an empirical example using a real-life sample from a publicly available database of the Nursing Home Compare [6], a national survey conducted by the Centers for Medicare and Medicaid Services (CMS) in the United States.

## Methods

### PSM assumptions

Suppose one has *N* units. In addition to a response value *Y*_*i*_, each of *N* units has a covariate value vector **X**_*i*_ = (*X*_*i*1_, …, *X*_*iK*_)′, where *i* = 1, …, *N*, and *K* is the number of covariates. Let *T*_*i*_ be the treatment condition. *T*_*i*_ = 1 indicates that unit *i* is in the treatment group and *T*_*i*_ = 0 the comparison group. Rosenbaum and Rubin [1] defined a propensity score for unit *i* as the probability of the unit being assigned to the treatment group, conditional on the covariate vector **X**_*i*_; that is,

1 $$ p\left({\mathbf{X}}_i\right)= Pr\left({T}_i=1\Big|{\mathbf{X}}_i\right). $$

PSM is based on the following two strong ignorability assumptions in treatment assignment [1]: (1) (*Y*_1*i*_, *Y*_0*i*_) ⊥ *T*_*i*_ | **X**_*i*_; and (2) 0 < *p*(**X**_*i*_) < 1. The first assumption states a condition that treatment assignment *T*_*i*_ and response (*Y*_1*i*_, *Y*_0*i*_) are conditionally independent, given **X**_*i*_; the second one ensures a common support between the treatment and comparison groups.

Rosenbaum and Rubin [1] further demonstrated in their Theorem 3 that ignorability conditional on **X**_*i*_ implies ignorability conditional on *p*(**X**_*i*_); that is,

2 $$ \left({Y}_{1i},{Y}_{0i}\right)\perp {T}_i\left|{\mathbf{X}}_i\Rightarrow \left({Y}_{1i},{Y}_{0i}\right)\perp {T}_i\right|p\left({\mathbf{X}}_i\right). $$

Thus, under the assumptions of the strong ignorability in treatment assignment, if a unit in the treatment group and a corresponding matched unit in the comparison group have the same propensity score, the two matched units will have, in probability, the same value of the covariate vector **X**_*i*_. Therefore, outcome analysis on the matched data after matching tends to produce unbiased estimates of treatment effects due to reduced selection bias through balancing the distributions of observed covariates between the treatment and comparison groups [1, 2, 7]. In practice, the logit of propensity score, *l*(**X**_*i*_) = ln{*p*(**X**_*i*_)/[1 – *p*(**X**_*i*_)]}, rather than the propensity score *p*(**X**_*i*_) itself, is commonly used because *l*(**X**_*i*_) has a better property of normality than does *p*(**X**_*i*_) [1].

### PSM methods

The basis of PSM is *nearest neighbor matching* [8], which matches unit *i* in the treatment group with unit *j* in the comparison group with the closest distance between the two units’ logit of their propensity scores expressed as follows:

3 $$ d\left(i,j\right)={ \min}_j\left\{\left|l\left({\mathbf{X}}_i\right)\hbox{--} l\left({\mathbf{X}}_j\right)\right|\right\}. $$

Alternatively, *caliper matching* [4] matches unit *i* in the treatment group with unit *j* in the comparison group within a pre-set caliper band *b*; that is,

4 $$ d\left(i,j\right)={ \min}_j\left\{\left|l\left({\mathbf{X}}_i\right)\hbox{--} l\left({\mathbf{X}}_j\right)\right|<b\right\}. $$

Based on Cochran and Rubin’s work [4], Rosenbaum and Rubin [8] recommend *b* equals 0.25 of the pooled standard deviation (*SD*) of the propensity scores. Austin [9] further asserted that *b* = 0.20 × *SD* of the propensity scores is the optimal caliper bandwidth.

Correspondingly, *Mahalanobis metric matching* (or Mahalanobis metric matching including the propensity score) and *Mahalanobis caliper matching* (or Mahalanobis metric matching within a propensity score caliper) [8] are two additional matching techniques similar to nearest neighbor matching and caliper matching, respectively, but use a diffident distance measure. In Mahalanobis metric matching, unit *i* in the treatment group is matched with unit *j* in the comparison group with the closest Mahalanobis distance measured as follows:

5 $$ d\left(i,j\right)={ \min}_j\left\{{D}_{ij}\right\}, $$

where *D*_*ij*_ = (**Z**_*i*_′ – **Z**j′)′S−1(**Z**i′ – **Z**j′), **Z**_•_ (• = *i* or *j*) is a new vector (**X**_•_, *l*(**X**_•_)), and **S** is the sample variance-covariance matrix of the vector for the comparison group. Mahalanobis caliper matching is a variant of Mahalanobis metric matching and it uses

6 $$ d\left(i,j\right)={ \min}_j\left\{{D}_{ij}<b\right\}, $$

where the selection of the caliper band *b* is the same as in caliper matching.

Data reduction after matching is a common and inevitable phenomenon in PSM. Loss of data in the comparison group seems a problem, but what we lose is unmatched cases that are assumed to potentially cause selection bias, and therefore, those unmatched units would have a negative impact on estimation of treatment effects. The matched data that may have a smaller sample size will, however, produce more valid (or less biased) estimates than do the original data. It is true that if we have small samples, which is not uncommon in medical research, PSM may not be applicable in such situations, but PSM is particularly useful in secondary data analysis on national databases such as the CMS data.

### PSM algorithms

All aforementioned PSM methods can be implemented by using either *greedy matching* or *optimal matching* algorithm [10]. Both matching algorithms usually produce similar matched data when the size of the comparison group is large; whereas optimal matching gives rise to smaller overall distances within matched units [11, 12]. All the matching techniques, either using greedy matching or optimal matching, are based on the distance between point estimates of propensity scores. The problem with this approach is that it is difficult to establish a meaningful criterion to evaluate the closeness of the matched units without knowing the standard errors of the estimated unit-specific propensity scores. Simply put, without knowing the standard errors of *l*(**X**_*i*_) and *l*(**X**_*j*_), we do not know if *l*(**X**_*j*_) in the comparison group is the best matched score with *l*(**X**_*i*_) in the treatment group. In other words, a score a little smaller than *l*(**X**_*j*_) might be a better matched one with *l*(**X**_*i*_); or conversely, *l*(**X**_*j*_) might be matched better with a score a little larger than *l*(**X**_*i*_).

Although caliper matching, one of the most effective matching methods [13–15], uses a caliper band to avoid “bad” matches, a caliper band is fixed (or unit-invariant) and cannot capture the unit-specific standard error of the estimated propensity score for each unit. Therefore, a new matching technique is needed for gauging standard errors of propensity scores.

### Interval matching

Interval matching extends caliper matching for accommodating the estimation error (or standard error) of the estimated propensity score by establishing a CI of the estimated propensity score for each unit. In interval matching, if the CI of a unit in the treatment group overlaps with that of one or more units in the comparison group, they are considered as matched units. Because the true distribution of propensity scores is unknown, the bootstrap [5] is utilized for obtaining a unit-specific CI for each unit. The bootstrap is a statistical method of assessing the accuracy (e.g., standard errors and CIs) of sample estimates to population parameters, based on the empirical distribution of sample estimates from random resamples of a given sample whose distribution is unknown.

Let {*X*_1_, …, *X*_*N*_} be a random sample of size *N* from an unknown distribution *F*; *θ*(*F*) is a parameter of interest. The specific procedure of the bootstrap for computing a CI of the parameter estimate, [${\widehat{\theta}}_{a/2}$ (*X*_1_, …, *X*_*N*_), ${\widehat{\theta}}_{1-a/2}$ (*X*_1_, …, *X*_*N*_)], where (1 - α) is the confidence level, consists of the following four steps:

1. Obtain a bootstrap sample {*X*_1_*, …, *X*_N_*} that is randomly resampled with replacement from the empirical distribution *F*_*N*_ represented by the original sample {*X*_1_, …, *X*_*N*_};
2. Calculate the parameter estimate $\widehat{\theta}$ (*X*_1_*, …, *X*_N_*) for the quantity *θ*(*F*_*N*_) = *θ*(*X*_1_, …, *X*_*N*_);
3. Repeat the same independent resampling-calculating scheme *B* times (typically 500 times), resulting in *B* bootstrap estimates $\widehat{\theta}$ (*X*_1_*^(*b*)^, …, *X*_N_*^(*b*)^), *b* = 1, …, *B*, which constitute an empirical distribution (or sampling distribution) of the estimate $\widehat{\theta}$ (*X*_1_, …, *X*_*N*_); and
4. Obtain the estimated CI of the parameter estimate, [${\widehat{\theta}}_{a/2}$ (*X*_1_, …, *X*_*N*_), ${\widehat{\theta}}_{1-a/2}$ (*X*_1_, …, *X*_*N*_)], by computing the (α/2)th and (1 – α/2)th percentiles of the sampling distribution, ${\widehat{\theta}}_{a/2}$ (*X*_1_*, …, *X*_N_*) and ${\widehat{\theta}}_{1-a/2}$ (*X*_1_*, …, *X*_N_*).

**Fig. 1:**
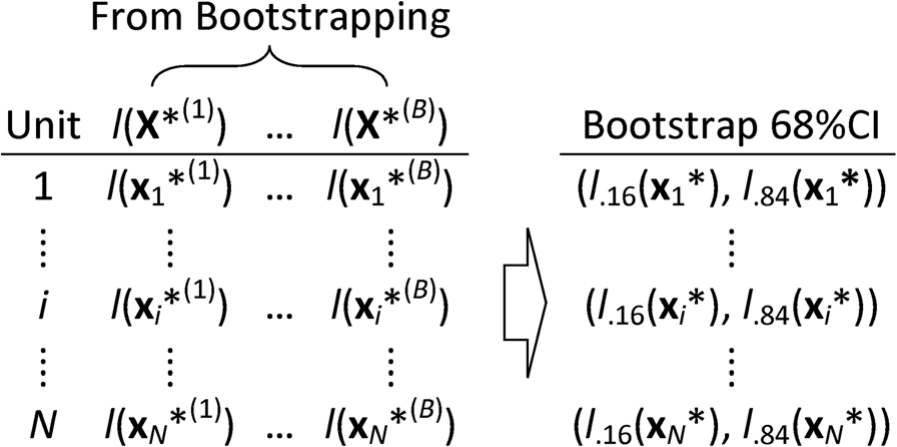
The procedure of obtaining bootstrap 68 %CIs of the logit of propensity scores

To obtain the bootstrap CIs for interval matching, one can simply follow the steps described above. First, conduct the bootstrap resampling *B* times on units in the sample data (*T*, **X**), where *T* is the indicator of the treatment conditions and **X** is the covariate value matrix (**X**_1_, …, **X**_*N*_)′, resulting in *B* bootstrap samples (*T*^(*b*)^, **X**^(*b*)^), where **X**^(*b*)^ = (**X**_1_*^(*b*)^, …, **X**_N_*^(*b*)^)′, *b* = 1, …, *B*. Second, a logistic regression (or other propensity score estimation model) is repeatedly applied to each of the *B* bootstrap samples, resulting in *B* propensity scores for each unit *i* (*i* = 1, …, *N*): *p*(**X**_i_*^(1)^), …, *p*(**X**_i_*^(*B*)^); then, their logit, *l*(**X**_i_*^(1)^), …, *l*(**X**_i_*^(*B*)^), are calculated. Last, for each unit *i*, a CI at certain confidence level (e.g., 68 %CI) is obtained by calculating the corresponding percentiles of the sampling distribution of the logit of *B* bootstrap propensity scores. Specifically, an estimated bootstrap 68 %CI for the logit of the propensity score of unit *i* would be [*l*_.16_(**X**_i_*), *l*_.84_(**X**_i_*)] (see Fig. 1 for an illustration).

Once a CI of the estimate of the logit of propensity score is obtained for each unit, interval matching can be conducted by examining whether the CI for a unit in the treatment group overlaps with that for one or more units in the comparison group. In other words, if the two CIs overlap; that is,

7 $$ \left[{l}_{.16}\left({\mathbf{X}}_i*\right),{l}_{.84}\left({\mathbf{X}}_i*\right)\right]\cap \left[{l}_{.16}\left({\mathbf{X}}_j*\right),{l}_{.84}\left({\mathbf{X}}_j*\right)\right]\ne \varnothing, $$

the two units are taken as matched units. In practice, one can do either 1:1 or 1:*K* interval matching. In 1:1 interval matching, one needs to take only one unit that has the closest distance, as defined by the matching method (e.g., Equation 3 for nearest neighbor matching and Equation 6 for Mahalanobis caliper matching), between the logit of the propensity scores among all the units in the comparison group whose CIs overlap with that of the unit in the treatment group. If there are two or more units in the comparison group within the overlap having the same closest distance, the program will randomly select one as the matched unit. In 1:*K* interval matching, one can simply take *K* closest units in the comparison group whose CIs overlap with that of the unit in the treatment group.

It is worth noting that using the logit of propensity score *l*(**X**_*i*_) is particularly important in interval matching because the distribution of logit *l*(**X**_*i*_) is more symmetric than the propensity score *p*(**X**_*i*_); therefore, interval matching based on logit *l*(**X**_*i*_) will be more balanced in terms of matching from both sides (left or right) of the distribution of logit *l*(**X**_*i*_).

## Results

The procedure of interval matching is illustrated in an empirical example that was stemmed from Lutfiyya, Gessert, and Lipsky’s comparative study [16]. They compared nursing home quality between rural and urban facilities using the CMS Nursing Home Compare data in 2010 on the past performance of all Medicare- and Medicaid-certified nursing homes in the United States [6]. The data were downloaded from the CMS Nursing Home Compare Website on more than 10,000 nursing homes with the geographical location (rural vs. urban) information extracted from the 2003 rural–urban county continuum codes developed by the Economic Research Service of the United States Department of Agriculture [17]. Quality ratings on nursing home performance were measured on three domains: health inspection, staffing, and quality measures [6]. An overall rating was also computed as a weighted average of the three domains. Lutfiyya, Gessert, and Lipsky [16] concluded that rural nursing home quality was not comparable to that of urban nursing homes with mixed findings: rural nursing homes had significantly higher quality ratings on the overall rating (*p* < .001) and health inspections rating (*p* < .001) than did urban nursing homes, but significantly lower on the quality measures rating (*p* < .001) than did urban nursing homes; while there was no significant difference in nursing staffing rating (*p* = .480) between rural and urban nursing homes.

The problem in Lutfiyya, Gessert, and Lipsky’s study [16] is that the geographical location (rural vs. urban) of nursing homes was not randomly assigned, and consequently, unbalanced background characteristics of nursing homes created potential selection bias between rural and urban nursing homes. Propensity score methods would be an appropriate technique to deal with this selection bias problem in such observational study.

### Data source

For illustration purposes only, the data used in this empirical example were a 50 % random sample from the same publicly available database, the CMS Nursing Home Compare in 2010. The sample data consisted of total *N* = 6,317 nursing homes (*n*_R_ = 1,990 rural nursing homes and *n*_U_ = 4,327 urban nursing homes) with 74 covariates of the ownership and size of nursing homes, qualification of nursing staff, and safety measures (see Additional file 1 for a full list of the 74 covariates). The 74 covariates were hypothesized to be related to the quality ratings and/or group assignment and, thus, all included in this empirical example. Due to the scope of this example and the space limit, general guidelines on covariate selection is not discussed here but available elsewhere [18].

It is also worth noting that due to the purpose of this example which is to illustrate the procedure of interval matching, replicating Lutfiyya, Gessert, and Lipsky’s study [16] of testing the difference in nursing home quality between rural and urban nursing homes was not the main focus of this example; instead, this example focused on evaluating the effectiveness of interval matching along with other commonly used PSM methods for reducing selection bias (or balancing covariates) between rural and urban nursing homes. Also, without loss of generality, 1:1 interval matching was illustrated; the present example can be easily extended to 1:*K* interval matching without any difficulty.

### Propensity score bootstrap CIs

Five hundred bootstrap samples were first resampled from the data using SAS® PROC SURVEYSELECT [19], and then for each of the 500 bootstrap samples, logistic regression of rural vs. urban nursing homes on the 74 covariates was conducted to obtain the probability (or the propensity score) of being a rural nursing home for each nursing home. There are some other propensity score estimation models, but without loss of generality, logistic regression was used in this example for illustration purposes only. Next, the logit of the propensity score for each nursing home was computed, and bootstrap 50 %, 68 %, and 95 %CIs of the logit for each nursing home were constructed by calculating the 25th percentile and the 75th percentile, the 16th percentile and the 84th percentile, and the 2.5th percentile and the 97.5th percentile, respectively, of the 500 bootstrap logit values. The purpose of computing the bootstrap CIs at different confidence levels was to examine the effect of the confidence level on the selection bias reduction in interval matching. Analogous to caliper bandwidth in caliper matching, the average of the half widths of the 6,317 bootstrap CIs was 0.20, ranging from 0.06 to 7.96 with a standard deviation of 0.19, for 50 %CIs; 0.29, ranging from 0.09 to 11.03 with a standard deviation of 0.29, for 68 %CIs; and 0.59, ranging from 0.20 to 26.35 with a standard deviation of 0.70, for 95 %CIs.

### Matching and evaluation of matching quality

The effectiveness of interval matching for reducing selection bias was evaluated along with the basic neighbor matching and the related caliper matching as well as other two commonly used matching methods, Mahalanobis caliper matching and optimal matching. All but optimal matching methods were implemented using a modified SAS® Macro based on Coca-Perraillon [20]. The optimal matching was conducted using an R package, *MatchIt* [12]. The pooled *SD* of the logit of the propensity scores *l*(**X**_*i*_) (*i* = 1, 2, …, 6,317) was 1.86; the caliper band for caliper matching in this example was *b* = 0.20 × *SD* = 0.20 × 1.86 = 0.37.

**Fig. 2:**
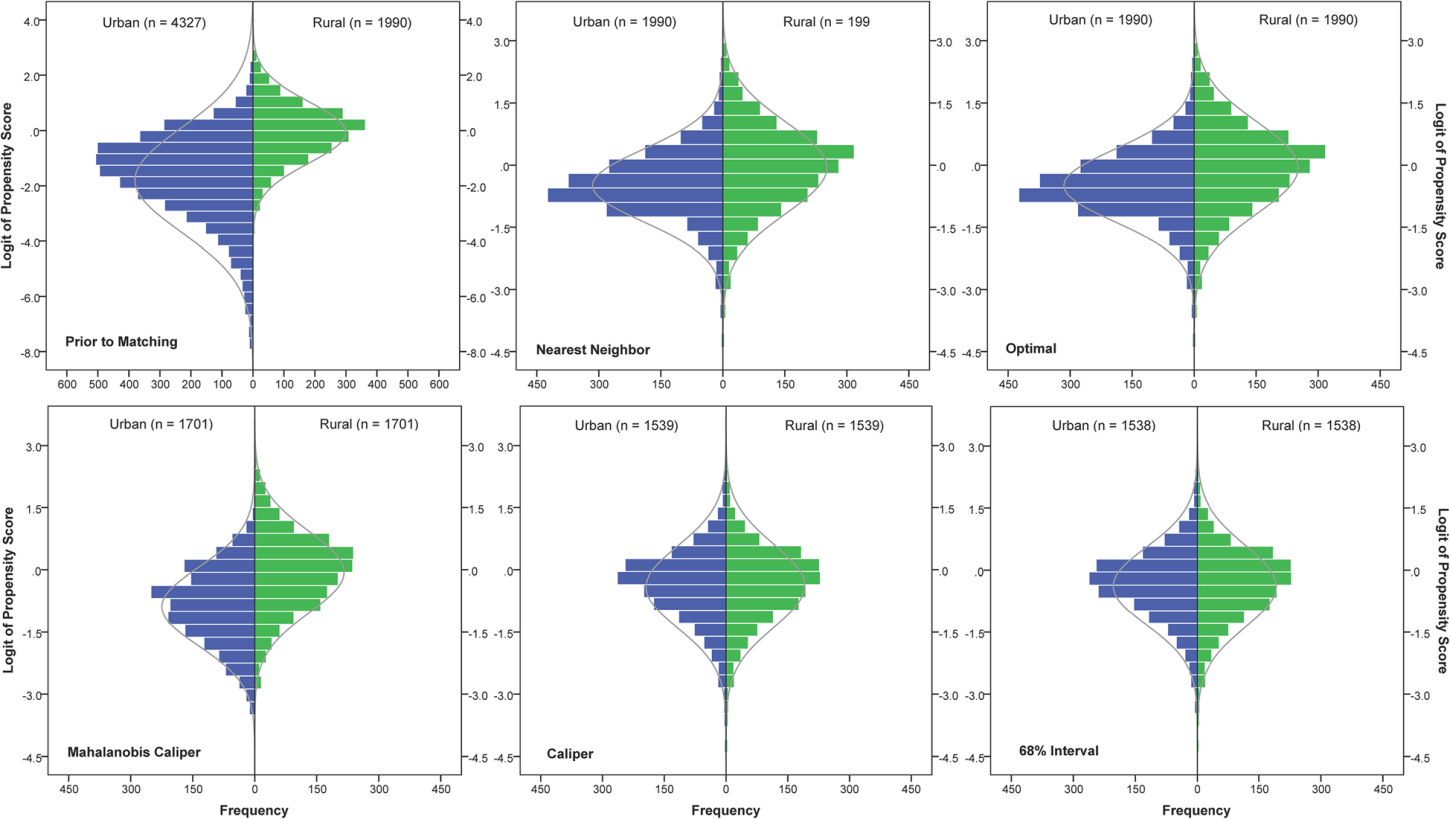
The distributions of the logit of propensity scores across the rural vs. urban nursing homes prior to and post matching

Figure 2 displays the distributions of the logit of propensity scores between the rural and urban nursing homes prior to and post matching. By visually inspecting the distributions of the logit of propensity scores, it can be seen that interval matching as well as caliper matching did better in balancing the distributions between the rural and urban nursing homes than did nearest neighbor matching, optimal matching, and Mahalanobis caliper matching. Three statistical criteria were also used to evaluate the effectiveness of the matching methods in balancing the distributions. They were the mean difference (or selection bias [*B*]), the standardized bias (*SB*), and the percent bias reduction (*PBR*).

The selection bias for each covariate *X*_*k*_ (*k* = 1, …, *K*) is the mean difference between the rural and urban nursing homes as follows:

8 $$ B={M}_1\left({X}_k\right)\hbox{--} {M}_0\left({X}_k\right), $$

where *M*_1_(*X*_*k*_) is the mean of the covariate for the rural nursing homes and *M*_0_(*X*_*k*_) is the mean of the covariate for the urban nursing homes. The *SB* associated with each covariate was defined by Rosenbaum and Rubin [8] as follows:

9 $$ SB=\frac{B}{\sqrt{\frac{V_1\left({X}_k\right)+{V}_0\left({X}_k\right)}{2}}}\times 100\%, $$

where *V*_1_(*X*_*k*_) is the variance of the covariate for the rural nursing homes and *V*_0_(*X*_*k*_) is the variance of the covariate for the urban nursing homes. According to Caliendo and Kopeinig [21], if the absolute *SB* is reduced to 5 % or less after matching, the matching method is considered effective in reducing selection bias. The *PBR* on the covariate was proposed by Cochran and Rubin [4] and it can be expressed as follows:

10 $$ PBR=\frac{\left|{B}_{prior\ to\  matching}\left|-\right|{B}_{post\  matching}\right|}{\left|{B}_{prior\ to\  matching}\right|}\times 100\%. $$

Note that the original expression of *PBR* in the literature [2, 4, 22, 23] did not impose the absolute values for *B*; here *PBR* (Equation 10) includes the absolute values to make the criterion more meaningful because both positive and negative *B*s indicate unbalanced distributions of the covariate.

Table 1 displays a summary of selection bias prior to matching and bias reduction post matching (see Additional file 2 for selection bias prior to matching and bias reduction post matching for all 74 covariates). From Table 1, we can see that selection bias prior to matching was evident in that the average of the 74 absolute *SB*s was 16.22 %. In addition, the selection bias is also indicated by the severely unbalanced distributions of the logit of the propensity scores with *SB* = 78.73 %.

**Table 1:** A summary of selection bias prior to matching and bias reduction post matching

Matching Method	Sample Size	*SB* for Logit of PS (%)	*PBR* for Logit of PS (%)	Average of Absolute *SB* across 74 Covariates (%)	Average of *PBR* across 74 Covariates (%)
Prior to Matching	*n*_R_ = 1990 *n*_U_ = 4327	78.73	—	16.22	—
Post Matching					
Nearest Neighbor	*n*_R_ = *n*_U_ = 1990	30.23	75.21	4.84	53.32
Optimal	*n*_R_ = *n*_U_ = 1990	30.23	75.21	4.91	55.15
Mahalanobis Caliper	*n*_R_ = *n*_U_ = 1701	61.04	51.79	10.16	25.70
Caliper	*n*_R_ = *n*_U_ = 1539	1.96	98.41	1.00	76.78
50 % Interval	*n*_R_ = *n*_U_ = 1483	−2.89	97.69	1.43	76.50
68 % Interval	*n*_R_ = *n*_U_ = 1538	−0.46	99.64	1.25	79.24
95 % Interval	*n*_R_ = *n*_U_ = 1713	9.52	92.55	1.33	79.12

**Fig. 3:**
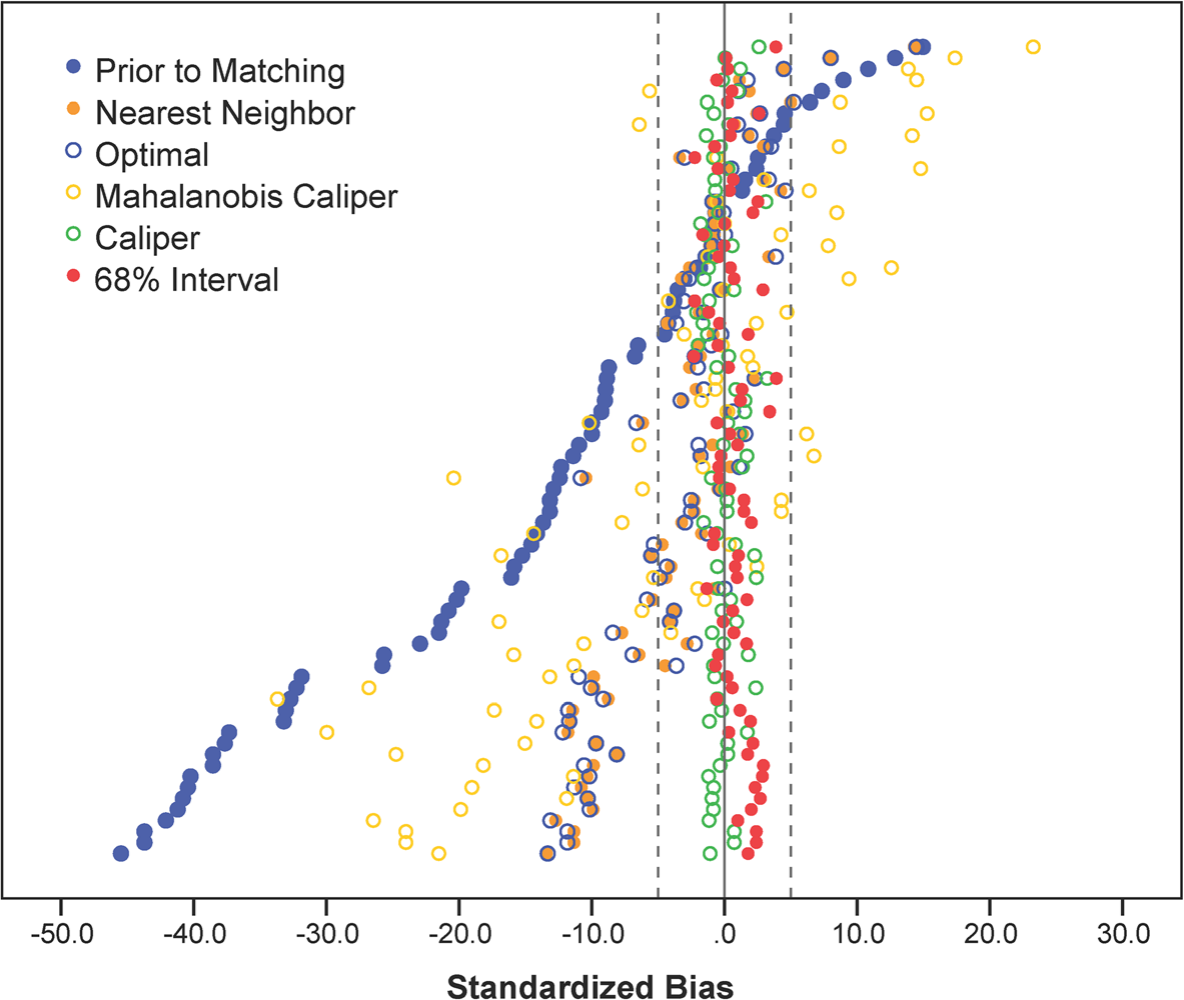
The standardized bias demonstrating the covariate balance prior to and post matching

The results of applying nearest neighbor matching, optimal matching, Mahalanobis caliper matching, caliper matching, and three interval matching methods are also presented in Table 1. First of all, the average of absolute *SB*s, and average *PBR*s across all 74 covariates demonstrated that the three interval matching methods as well as caliper matching were superior to all other matching methods by all means. Furthermore, by examining the statistical criteria for the logit of propensity scores—“arguably the most important variable” ([8], p. 36) in balancing the distributions of the covariates, the data suggested that 68 % interval matching outperformed caliper matching because the interval matching removed 99.64 % of the selection bias with remaining *SB* = −0.46 %, compared to 98.41 % for caliper matching (remaining *SB* = 1.96 %). In addition, this favorable phenomenon to 68 % interval matching was also echoed by the average *PBR* across all covariates ($\overline{PBR}$ = 79.24 %), compared to $\overline{PBR}$ = 76.78 % for caliper matching; only the average $\overline{SB}$ of 68 % interval matching was slightly larger than but comparable to that of caliper matching (1.25 % vs. 1.0 %). Individual covariate balancing is also summarized in a graphical display (see Fig. 3) of *SB*s prior to and post the five matching methods. It is clearly seen that both interval matching and caliper matching significantly reduced more selection bias than did other matching methods because all the *SB*s of interval matching and caliper matching were within 5 %; whereas a substantial amount of the *SB*s of other matching were larger than 5 %.

## Discussion

The present study used bootstrap CIs at 50 %, 68 %, and 95 % confidence levels in the empirical example which demonstrated that 68 %CIs performed the best among the three (see Table 1) and to some extent better than caliper matching. When the empirical distribution of the logit of the estimated propensity score is normally distributed, a 68 %CI will be a range of ±1 standard error away from the mean; whereas the caliper band in caliper matching uses 0.20 standard deviation of the logit of the propensity score. A higher level of percentage (i.e., confidence level) (>68 %) will lead to more possible matched units and a lower level of percentage (<68 %) will lead to more rigid matching and, thus, possible fewer matched units. In practice, researchers can determine what percentage of CI to use for accommodating a different size of a comparison group. In general, a smaller percentage of CI may be used for a larger comparison group. In addition, 500 bootstrap samples were used in the empirical example. If some units are not selected in a bootstrap sample, a larger number of bootstrap samples may be used to avoid the situation where few bootstrap propensity scores are obtained for the unit.

As a side note, the difference in nursing home quality between rural and urban homes were compared using the matched data with 68 % interval matching, and the results (see Table 2) are different from those of Lutfiyya, Gessert, and Lipsky’s study [16]. Specifically, Table 2 shows that rural nursing homes had lower quality ratings on all the ratings than urban nursing homes, but only quality measures rating was significant (*p* < .001).

**Table 2:** Means (standard deviations) of nursing home quality ratings and independent samples *t*-test on the matched data with 68 % interval matching (*n*_rural_ = *n*_urban_ = 1,538)

Nursing Home Quality Rating	Geographical Location		*t*	*p*
	Rural	Urban		
*Overall rating*	3.06(1.30)	3.12(1.31)	−1.188	.235
Health inspections rating	2.90(1.26)	2.91(1.31)	−0.126	.899
Nurse staffing rating	2.98(1.22)	3.01(1.21)	−0.740	.459
Quality measures rating	3.14(1.23)	3.30(1.20)	−3.535	< .001

## Conclusions

The normal procedure of current PSM is to match each unit in the treatment group with one or more units in the comparison group based on the distance between the point estimates of propensity scores. Unfortunately, the point estimates cannot capture estimation errors (or standard errors) of propensity scores. The present study proposed interval matching using bootstrap CIs for accommodating unit-specific standard errors of (the logit of) propensity scores. Interval matching’s approach methodologically sounds more meaningful than its competing matching methods because interval matching develop a more “scientific” criterion for matching units using confidence intervals.

**Fig. 4:**
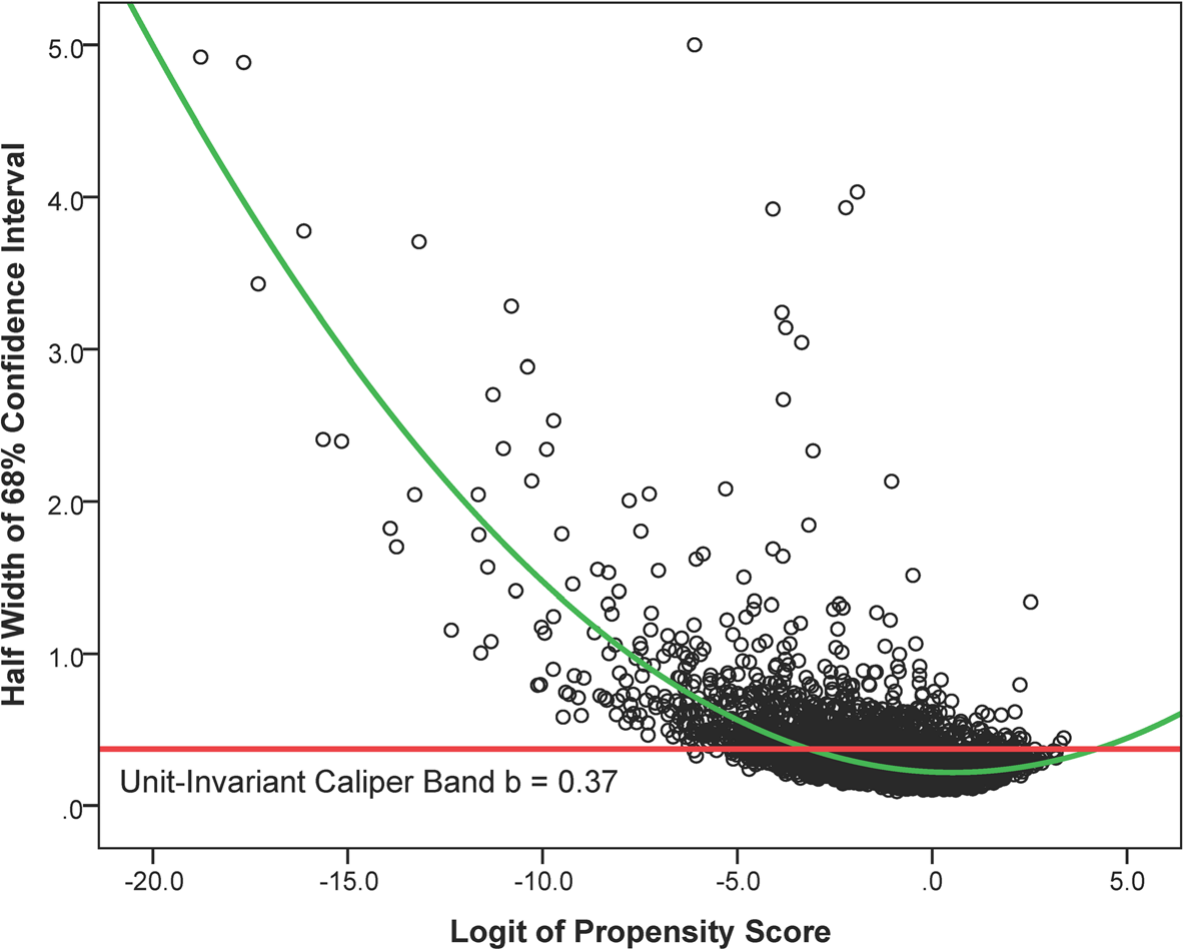
The curve-linear relationship (green) between the half width of the bootstrap 68 %CI and the logit of the propensity score, compared with the unit-invariant caliper band (red)

Besides accommodating standard errors of propensity scores using confidence intervals, interval matching has another methodologically sound property. That is, CIs of the logit of estimated propensity scores in relatively sparse areas where it is less likely to find matched units would be wider than those in the area with more dense data where it is more likely to find matched units. This curve-linear relationship between the width of CIs and the density of the distribution of the logit of propensity scores may lead to more matched units in sparse areas to balance out the area with more dense data (see Fig. 4); whereas caliper matching has a fixed caliper bandwidth (e.g., *b* = 0.37 for this empirical example) for all the values of the logit of propensity scores regardless the density of the distribution of the logit of propensity scores.

Because these beneficial properties of interval matching, the empirical example demonstrated that interval matching is not only a viable alternative to caliper matching, but also produced promisingly more balanced data than did all other matching methods including caliper matching.

It is true that the computation in interval matching is somewhat more labor intensive than that in other PSM methods. However, it should not be a problem in today’s fast computing technology, which makes the encouraging results in interval matching overweigh its intensive computation.

In future research, we would like to further explore the effectiveness of interval matching on reducing selection bias in a simulation study by creating different scenarios, such as 1:*K* matching, matching with replacement, sample size ratio of treatment group to comparison group, and size of common support between treatment and comparison groups. In addition to the effectiveness of interval matching on reducing selection bias, it would be also desirable to examine the effectiveness of interval matching on reducing estimation bias for treatment effects under various scenarios, comparing with some other matching techniques mainly for bias reduction in estimating treatment effects, such as full matching, subclassification, kernel matching (or difference-in-deference matching), as well as different propensity score estimation models.

In sum, interval matching possess sound methodological properties and is a promisingly better alternative tool for reducing selection bias in making causal inference from observational studies, especially helpful in secondary data analysis on national databases such as the CMS data as demonstrated in the empirical example.

## Additional files

Additional file 1:
**A list of the 74 covariates in the example data from the CMS nursing home compare database.** (DOCX 18 kb) Additional file 2:
**Selection bias prior to matching and bias reduction post matching for all 74 covariates.** (XLS 62 kb)

## Abbreviations

B: mean difference (or selection bias)
CI: confidence interval
CMS: the Centers for Medicare and Medicaid Services
PBR: percent bias reduction
PSM: propensity score matching
SB: standardized bias
SD: standard deviation
