# Supplementary material for: Propensity score interval matching: using bootstrap confidence intervals for accommodating estimation errors of propensity scores
Source: BMC Med Res Methodol. 2015 Jul 28;15:53. doi: 10.1186/s12874-015-0049-3 (PMC4517543; doi:10.1186/s12874-015-0049-3)
Supplement: Additional file 1: — A list of the 74 covariates in the example data from the CMS nursing home compare database. (DOCX 18 kb) [file 12874_2015_49_MOESM1_ESM.docx]

**Additional file 1. A list of the 74 covariates in the example data from the CMS nursing home compare database.** ^a^Data source: *prov2010_nodupkits.xls*. ^b^IN_HOSPITAL = PROVBASE. ^c^Data source: *ProviderInfo.xlsx*. ^d^W_A_C_SCORE = WEIGHTED_ALL_CYCLES_SCORE.

| **Covariate** | **Label** |
| --- | --- |
| FOR_PROFIT^a^ | Control (1-3) |
| NON_PROFIT^a^ | Control (4-6) |
| IN_HOSPITAL^a, b^ | If provider is in a hospital, either ‘Y’ or ‘N’ |
| CHAIN^a^ | If provider is in a chain, either ‘Y’ or ‘N’ |
| DEFNUM^a^ | Total number of deficiencies on current survey, health and life safety code |
| BEDTOT^a^ | Total number of beds, including non-participating |
| BEDCERT^a^ | Total Medicare and or Medicaid certified beds |
| RESTOT^a^ | Total number of residents in certified beds |
| CENSMCRE^a^ | Number of residents who are Medicare recipients |
| BEDCARE^a^ | Number of Medicare certified beds |
| CENSMCD^a^ | Number of residents who are Medicaid recipients |
| CENSOTH^a^ | Number of residents not Medicare or Medicaid recipients |
| SNF_DC^a^ | ‘02’-Skilled Nursing Facility/Nursing Facility - Dually Certified |
| SNF_DP^a^ | ‘03’-Skilled Nursing Facility/Nursing Facility - Distinct Parts |
| SNF^a^ | ‘04’-Skilled Nursing Facilities |
| NF^a^ | ‘10’-Nursing Facilities (Medicaid only) |
| CURHDEF^a^ | Total number of health deficiencies on current survey |
| CURLDEF^a^ | Total number of life safety code defs on current survey |
| TRANSFER^a^ | Transferring - number of residents dependent on staff |
| TOILET^a^ | Toilet use - number of residents dependent on staff |
| EATING^a^ | Eating - number of residents dependent on staff |
| CONTIN^a^ | Continence - number of residents occasionally or frequently incontinent of bladder |
| BEDFAST^a^ | Mobility - number of residents who are bedfast most or all of the time |
| CONTRACT^a^ | Mobility - number of residents with contractures |
| PSORES^a^ | Skin integrity - number of residents with pressure sores, excluding stage 1 |
| RESTRAIN^a^ | Mobility - number of physically restrained residents |
| WEIGHT^a^ | Other - number of residents with unplanned significant weight loss/gain |
| BEHAVE^a^ | Mental status - number of residents with behavioral symptoms |
| MEDAID_FT^a^ | Medication aide full-time as FTEs |
| MEDAID_PT^a^ | Medication aide part-time as FTEs |
| MEDAID_CT^a^ | Medication aide contract time as FTEs |
| RNDON_FT^a^ | Director of nursing full-time as FTEs |
| RNDON_PT^a^ | Director of nursing part-time as FTEs |
| RNDON_CT^a^ | Director of nursing contract time as FTEs |
| NRSADM_CT^a^ | Nurses w/ administrative duties full-time as FTEs |
| NRSADM_FT^a^ | Nurses w/ administrative duties part-time as FTEs |
| NRSADM_PT^a^ | Nurses w/ administrative duties contract time as FTEs |
| RN_FT^a^ | Registered nurse full-time as FTEs |
| RN_PT^a^ | Registered nurse part-time as FTEs |
| RN_CT^a^ | Registered nurse contract time as FTEs |
| VOC_FT^a^ | LPN/LVN full-time as FTEs |
| VOC_PT^a^ | LPN/LVN part-time as FTEs |
| VOC_CT^a^ | LPN/LVN contract time as FTEs |
| AID_FT^a^ | Nurse aide full-time as FTEs |
| AID_PT^a^ | Nurse aide part-time as FTEs |
| AID_CT^a^ | Nurse aide contract time as FTEs |
| AIDTRN_FT^a^ | Nurse aides in training full-time as FTEs |
| AIDTRN_PT^a^ | Nurse aides in training part-time as FTEs |
| AIDTRN_CT^a^ | Nurse aides in training contract time as FTEs |
| RESGROUP^a^ | The provider has resident group, either 'y' or 'n' |
| FAMGROUP^a^ | The provider has a family group, either 'y' or 'n' |
| CHOW_COUNTER^a^ | The number of times a change of ownership has taken place for a particular provider |
| SFF^c^ | Special Focus Facility |
| AIDHRD^c^ | Reported CNA Staffing Hours per Resident per Day |
| VOCHRD^c^ | Reported LPN Staffing Hours per Resident per Day |
| RNHRD^c^ | Reported RN Staffing Hours per Resident per Day |
| TOTLICHRD^c^ | Reported Licensed Staffing Hours per Resident per Day |
| TOTHRD^c^ | Reported Total Nurse Staffing Hours per Resident per Day |
| PTHRD^c^ | Reported Physical Therapist Staffing Hours per Resident Per Day |
| EXP_AIDE^c^ | Expected CNA Staffing Hours per Resident per Day |
| EXP_LPN^c^ | Expected LPN Staffing Hours per Resident per Day |
| EXP_RN^c^ | Expected RN Staffing Hours per Resident per Day |
| EXP_TOTAL^c^ | Expected Total Nurse Staffing Hours per Resident per Day |
| ADJ_AIDE^c^ | Adjusted CNA Staffing Hours per Resident per Day |
| ADJ_LPN^c^ | Adjusted RN Staffing Hours per Resident per Day |
| ADJ_RN^c^ | Adjusted LPN Staffing Hours per Resident per Day |
| ADJ_TOTAL^c^ | Adjusted Total Nurse Staffing Hours per Resident per Day |
| W_A_C_SCORE^c, d^ | Total Weighted Health Survey Score |
| INCIDENT_CNT^c^ | Number of Facility Reported Incidents |
| CMPLNT_CNT^c^ | Number of Substantiated Complaints |
| FINE_CNT^c^ | Number of Fines |
| FINE_TOT^c^ | Total Amount of Fines in Dollars |
| PAYDEN_CNT^c^ | Number of Payment Denials |
| T_PENLTY_CNT^c^ | Total Number of Penalties |
